# Helically twisted photonic crystal fibres

**DOI:** 10.1098/rsta.2015.0440

**Published:** 2017-02-28

**Authors:** P. St.J. Russell, R. Beravat, G. K. L. Wong

**Affiliations:** Max Planck Institute for the Science of Light, Staudtstrasse 2, 91058 Erlangen, Germany

**Keywords:** chirality, photonic crystal fibres, optical activity, orbital angular momentum, helical Bloch waves, Faraday effect

## Abstract

Recent theoretical and experimental work on helically twisted photonic crystal fibres (PCFs) is reviewed. Helical Bloch theory is introduced, including a new formalism based on the tight-binding approximation. It is used to explore and explain a variety of unusual effects that appear in a range of different twisted PCFs, including fibres with a single core and fibres with *N* cores arranged in a ring around the fibre axis. We discuss a new kind of birefringence that causes the propagation constants of left- and right-spinning optical vortices to be non-degenerate for the same order of orbital angular momentum (OAM). Topological effects, arising from the twisted periodic ‘space’, cause light to spiral around the fibre axis, with fascinating consequences, including the appearance of dips in the transmission spectrum and low loss guidance in coreless PCF. Discussing twisted fibres with a single off-axis core, we report that optical activity in a PCF is opposite in sign to that seen in a step-index fibre. Fabrication techniques are briefly described and emerging applications reviewed. The analytical results of helical Bloch theory are verified by an extensive series of ‘numerical experiments’ based on finite-element solutions of Maxwell's equations in a helicoidal frame.

This article is part of the themed issue ‘Optical orbital angular momentum’.

## 1. Introduction

**Figure 1.**
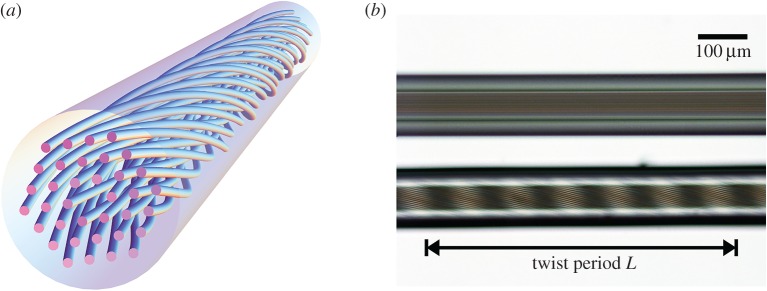
(*a*) Sketch of a twisted single-solid-core PCF. (*b*) Side-view photograph of a twisted PCF. Six moiré-like fringes (the six facets of the photonic crystal cladding) correspond to one twist period *L*. (Online version in colour.)

The behaviour of light in chiral structures continues to be a subject of great fundamental interest, and many applications are emerging in the various subfields of photonics. Examples include optical activity in biological molecules, lasing in cholesteric liquid crystals [1], chiral metamaterials [2], multielement helical structures (written by fs-laser-machining) that act like topological insulators [3] and twisted photonic crystal fibres (PCFs)—the topic of this review. Chiral materials typically consist of many identical chiral molecules either randomly oriented in solution, or ordered arrays of chiral units such as molecules or nanostructured elements. In contrast, a twisted PCF consists of a single uniaxial chiral unit, containing one or more light-guiding cores, that is infinitely extended in the third dimension the direction of the twist (figure 1). In its most common form, PCF consists of a hexagonal array of hollow microchannels running along the length of a strand of glass approximately 100 µm thick. When subjected to a continuous twist along its length, it has the appearance of a ‘multihelix’ (table 1).

**Table 1.** Nomenclature.

*a_n_*	amplitude of the field in the *n*th core
*_α_*	twist rate in rad m^−1^
*B*_C_	circular birefringence
*b_m_*	amplitude of *m*th Bloch harmonic
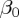	propagation constant in untwisted core
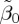	effective axial propagation constant in twisted PCF
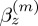	axial propagation constant of *m*th azimuthal harmonic of helical Bloch wave
*ε*_0_	average dielectric constant of periodically modulated ring
*ε*_1_	amplitude of sinusoidal dielectric constant modulation in ring
γ	eigenvalue correction to *β*_0_ in twisted PCF
*γ*_rot_	rotation of linear polarization in twisted PCF
*κ*	coupling constant between adjacent cores (tight-binding analysis)
*κ*_h_	coupling constant between adjacent Bloch harmonics (expansion in OAM orders)
*L*	period of helical twist
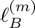	OAM order of *m*th Bloch harmonic in an untwisted fibre
*ℓ*^(*m*)^	OAM order of *m*th harmonic of helical Bloch mode in twisted fibre
*m*	Order of *m*th Bloch harmonic
*n*_a_	refractive index of material in hollow channels
*n*_c_	refractive index of core mode(s)
*n*_s_	refractive index of glass
*n*_SM_	refractive index of fundamental space-filling mode in cladding
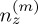	axial refractive index of *m*th azimuthal harmonic of helical Bloch wave
*N*	number of cores in the ring
OAM	orbital angular momentum
PCF	photonic crystal fibre
*P*(**r**)	periodic function of a Bloch mode
*q*(λ)	optical rotation angle as a fraction of the structural twist angle for PCF with on-axis core
*ψ*	local tilt angle of the hollow channels and glass strands in the twisted cladding
(*ρ*, *ϕ*)	radial and azimuthal coordinates
*ρ*_co_	radial position of off-axis core
(*x*, *y*, *z*)	Cartesian coordinates, *z* pointing along the fibre axis

The propagation of light in helically twisted waveguides has been the subject of many theoretical investigations over the past decade, for example studies of the creation of optical vortices in multihelicoidal fibres [4], the analysis of Bloch dynamics in helical waveguide arrays [5] and optical activity in multihelicoidal fibres [6].

Twisted optical fibres, drawn from a spinning preform, have been studied since at least the early 1980s. Interest in them stemmed initially from the need to reduce the residual birefringence of circularly symmetric step-index fibres, and thereby eliminate polarization mode dispersion in optical telecommunications [7,8]. If, on the other hand, the core is twofold symmetric, i.e. it is linearly birefringent, then it will support elliptically polarized eigenmodes when spun into a chiral structure. Such modes have been used in current and magnetic field sensing based on the Faraday effect [9,10].

In the mid-1980s, Fujii & Hussey [11] showed theoretically that a twisted fibre with an azimuthally segmented core would exhibit pure circular birefringence. This suggestion was not followed up experimentally, presumably owing to the difficulty of manufacturing such a structure. With the advent of PCF, this difficulty has been circumvented, and circularly birefringent twisted PCFs, exhibiting robust optical activity, have recently been reported [12]. Theoretical analysis, based on symmetry properties and perturbation theory, indicates that both spin and orbital angular momentum (OAM) play a role in this phenomenon [13].

The paper is organized as follows. First, we discuss theoretical aspects, starting with numerical techniques for solving Maxwell's equations in a helicoidal frame (§2). As well as allowing direct comparison with experimental results, the resulting ‘numerical experiments’ provide a reality check for the analytical theories that we then go on to develop, based on the concept of helical photonic Bloch waves. Using helical Bloch theory, we discuss a new kind of birefringence that causes the propagation constant of left and right-spinning optical vortices (modes carrying OAM [14,15]) to be non-degenerate for the same OAM order (§3). The next topic is topological effects in the twisted periodic cladding ‘space’, which causes the geodesics of the light to spiral around the fibre axis, with fascinating consequences, including the appearance of dips in the transmission spectrum corresponding to ring-like optical vortices in the cladding (§4). In §5, we discuss why the optical activity in a twisted PCF with an on-axis core is opposite in sign to that seen in a twisted step-index fibre with an off-axis core. Fabrication techniques are described in §8, and §7 deals with a number of applications of twisted PCFs. Conclusions are drawn in §8.

## 2. Numerical modelling in a helicoidal frame

The most natural coordinate frame for a continuously twisted PCF is helicoidal: a non-orthogonal curvilinear system in which the helicoidal coordinates (*ξ*_1_, *ξ*_2_, *ξ*_3_) are related to the Cartesian coordinates (*x*, *y*, *z*) as follows:
2.1
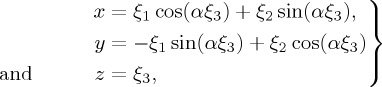

where *z* points along the fibre axis, and *α* is the twist rate in rad m^−1^. Maxwell's equations take the same form in any coordinate system [16], but when the *z*-dependent material permittivity and permeability tensors [*ε*] and [*μ*] in the laboratory frame are transformed into the helicoidal frame, they become axially invariant, developing off-diagonal elements as follows:
2.2
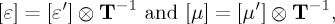

where [*ε*′] and [*μ′*] are the axially invariant permittivity and permeability tensors in the twisted frame, and **T** is the transformation matrix given by [17]
2.3
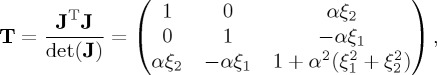

where **J** is the Jacobian of the Cartesian (*x*, *y*, *z*) to helicoidal (*ξ*_1_, *ξ*_2_, *ξ*_3_) transformation. **T** is independent of the axial coordinate *ξ*_3_, which turns the three-dimensional problem into a two-dimensional one and greatly simplifies the numerical calculations.

## 3. Helical Bloch waves: tight-binding approximation

The optical Bloch waves of any untwisted periodic structure are described by the product of a periodic function *P*(**r**) (with periodicities that match the structure) and a term representing the phase progression of the Bloch wave: *P*(**r**)exp i**k**_B_ · **r** [18]. A convenient physical picture for the modes guided in a helical PCF can be constructed by generalizing Bloch's theorem, so that the periodic function follows the twist, taking the form *P*(*ρ*,*ϕ *− *αz*), where *ρ* is the radial coordinate and *ϕ* is the azimuthal angle [19]. The Bloch waves can then be calculated analytically, using an expansion in terms of azimuthal harmonics of OAM order *ℓ*^(*m*) ^= *ℓ*^(0)^ + *Nm*, as described in [19] (see §3d). Here, we develop an alternative approach, one that uses the tight-binding approximation in a ring of *N* coupled cores.

### (a) Untwisted case

**Figure 2.**
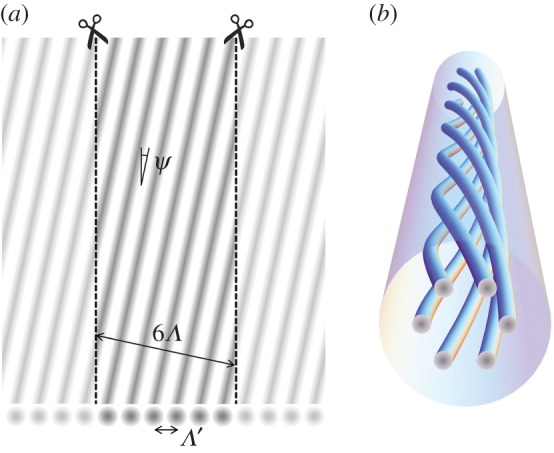
(*a*) Slanted array of parallel waveguides on a plane. (*b*) Six-core helical structure that results when six periods are cut out and wrapped around a cylinder. (Online version in colour.)

Scalar wave propagation in an *untwisted* ring of *N* guiding cores (*ψ* = 0 in figure 2) is governed by the master equation
3.1
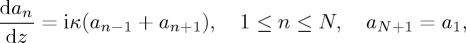

where *κ* is the coupling constant and nearest-neighbour coupling is assumed. The quantity *a_n_* is the amplitude of the mode in the *n*th core. Applying Bloch's theorem yields the dispersion relation
3.2
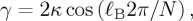

where *ℓ*_B_ is the angular Bloch wavevector in the azimuthal direction (with units of rad rad^−1^), and *γ* is the correction to the axial propagation constant *β*_0_ of an isolated core. Note that equation (3.2) is valid equally for an infinite array of parallel waveguides on a flat plane (*N* → ∞). In the ring, however, *ℓ*_B_ becomes discretized, taking only integer values 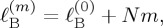 where 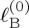 is the principal OAM order and 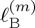 is the OAM order of the *m*th harmonic of the Bloch wave.

### (b) Twisted case

In a *twisted* fibre, the Bloch waves become helical, and can be written in the modified form
3.3
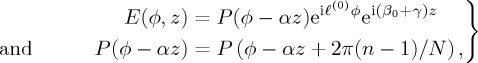

where *ϕ* is the azimuthal angle, *P*(*ϕ *− *αz*) is a periodic function that repeats *N* times in 2*π*, spinning as *z* increases, and 1 < *n* < *N* is the integral sector number. The principal OAM order in the plane normal to the fibre axis is *ℓ*^(0)^, and equals 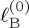 only in the untwisted structure.

The dispersion relation in the twisted fibre may be derived once it is realized that an *N*-period-wide strip of a tilted array of stripe waveguides on a flat surface, when wrapped around a cylinder, forms a replica of a twisted PCF with *N* cores in a ring (see figure 2 for the six-core case).

**Figure 3.**
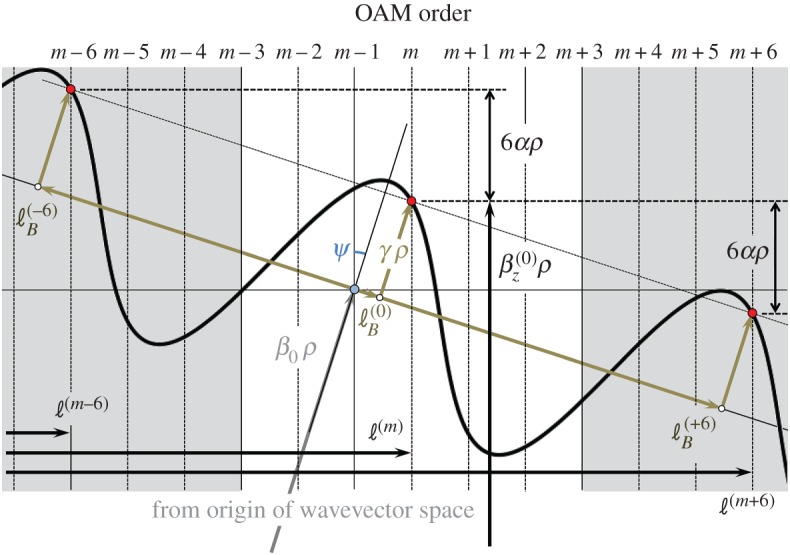
Slanted dispersion surface for six-core PCF in figure 2 illustrates the coordinate rotation into a system parallel to the fibre axis. (Online version in colour.)

All that is needed, then, to find the dispersion relation in the twisted structure is a coordinate rotation through tilt angle *ψ* = arctan(*αρ*), where *ρ* is the distance from the fibre axis to the satellite cores (figure 3).

After applying this rotation the following expressions result
3.4*a*
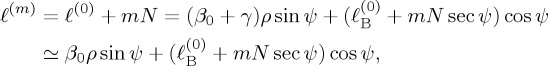

3.4*b*
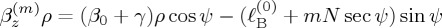

3.4*c*
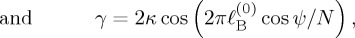

where the approximation in equation (3.4*a*) follows from *γ* sin *ψ* being small compared with all the other terms; note also that *N* is now aligned with the azimuthal direction, not with an axis perpendicular to the waveguides, so that an additional cos *ψ* factor is needed in the argument of the cosine in the expression for *γ*. Solving equation (3.4*a*) for 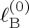 and substituting into equation (3.4*b*) yield an analytical expression for 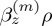

3.5
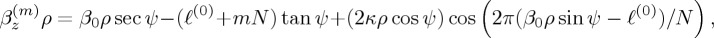


which, with the approximations sin *ψ *≈ tan *ψ *= *αρ ≪ 1*, taking terms up to order (*αρ*)^2^ and assuming that *κρ* is of the same order as *αρ*, becomes
3.6
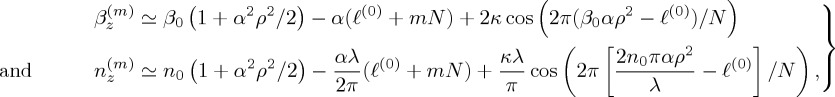

where 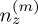 is the refractive index of the *m*th Bloch harmonic, *n*_0_ is the refractive index of the mode in an isolated core and λ is the vacuum wavelength.

### (c) Orbital angular momentum birefringence

Equation (3.6) shows that the splitting in index between positive and negative *ℓ*^(0)^ is approximately *ℓ*^(0)^*αλ*/*π*, assuming that the contribution from the last term is small. It also predicts a topological increase in base refractive index that is proportional to the square of the radius (the first term). Note also that the index difference between successive harmonics is *Nαλ*/(2*π*).

**Figure 4.**
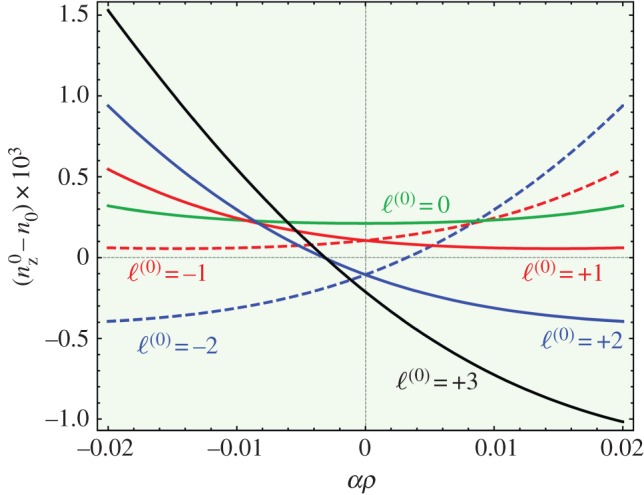
Modal refractive indices of helical Bloch modes with different OAM orders, calculated using equation (3.6) for *κρ*** **=** **0.005, *ρ*/λ** **=** **7.5, *N**** ***=** **6, *m**** ***=** **0 and *n*_0_** **=** **1.44*.* Note the splitting in refractive index between modes with principal OAM order ± 2 and ± 1, which scales linearly with *α* (see text). The *ℓ*^(0)^ =*** ***0 and +3 modes carry only very small amounts of OAM, the *ℓ*^(0) ^= 0 mode having the highest, and the *ℓ*^(0) ^= +3 mode the lowest, refractive index. (Online version in colour.)

The expression for the modal refractive indices in equation (3.6) has the interesting consequence that modes of the same principal OAM order *ℓ*^(0)^, but opposite sign, are non-degenerate in the twisted PCF (figure 4). This represents a new kind of birefringence that inhibits scattering from a left-spinning OAM mode into a right-spinning one—the twisted fibre behaves like a topological insulator.

### (d) Expansion in terms of orbital angular momentum harmonics

Further insights may be gained by expanding in terms of OAM harmonics, an approach that is closely related to the plane-wave expansion method commonly used to calculate the photonic band structure in periodic structures. It involves representing both the electromagnetic field and the dielectric constant as complex Fourier series, substituting these into Maxwell's equations and solving for the amplitudes of each harmonic of the Bloch wave [19]. A thin annular waveguide of radius *ρ* that is azimuthally periodic and axially twisted can described by the dielectric constant
3.7
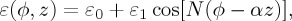

whereas before there are *N* periods around the circle, and 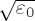 is the modal refractive index of the fundamental waveguide mode. The field Ansatz for the helical Bloch waves can then be written as
3.8
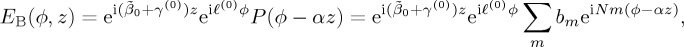

where 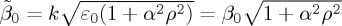 is the effective average axial propagation constant (taking account of the fact that the light is spiralling around the axis), *k* is the vacuum wavevector and *b_m_* is the complex amplitude of the *m*th Bloch harmonic. Note that the azimuthal interference pattern created by any two of the harmonics in the summation will rotate at exactly the same rate as the fibre twist, as required.

Inserting (3.7) and (3.8) into Maxwell's equations in cylindrical coordinates with no radial term
3.9
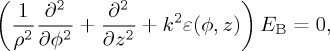

one then applies the standard condition that the sums of coefficients of terms with identical rates of phase progression must each independently equal zero [20]. This leads, after some algebra, to a set of homogeneous linear equations in the amplitudes *a_n_*
3.10
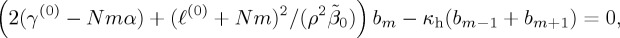

where the interharmonic coupling constant is defined by 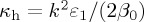 and it is assumed that 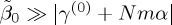 for values of *m* within the required truncation range. The truncated equation set can then be solved numerically for the dispersion relation *γ*^(0)^(*ℓ*^(0)^) and the eigenmodal shapes of the Bloch waves. The propagation constant of the *m*th harmonic is then 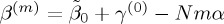.

**Figure 5.**
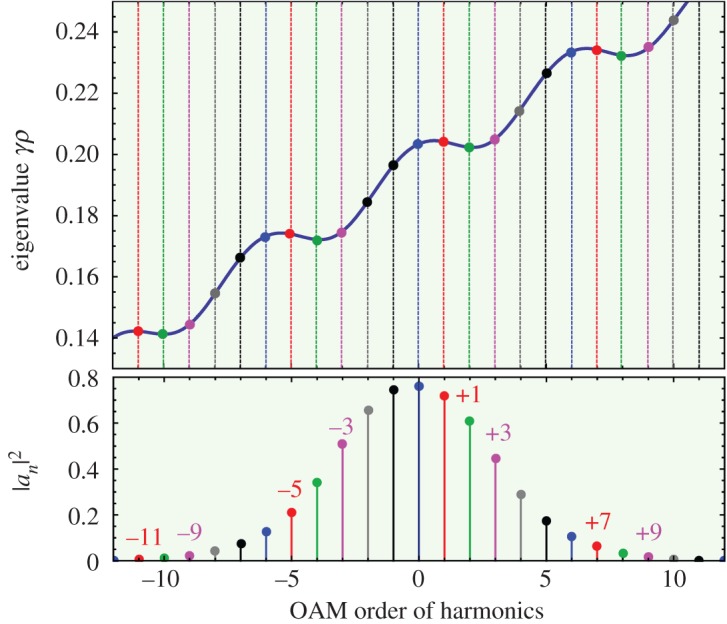
(*a*) Wavevector diagram for a ring of six coupled cores, plotted over four Brillouin zones. It was obtained by solving the plane-wave expansion in [19] for 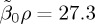, *αρ *= 0.0038 and *κ*_h_*ρ* = 0.1, including seven harmonics. Its shape is identical in each Brillouin zone (this ensures that every harmonic of the helical Bloch wave shares the same group velocity), and as predicted by equation (3.6), it is tilted at an average slope *αρ* (this ensures that the field pattern rotates with the helical structure). (*b*) The normalized strengths of each harmonic. The numbers correspond to the harmonic orders *m* of anticlockwise-spinning (violet) and clockwise-spinning (red) helical Bloch modes. (Online version in colour.)

An example is shown in figure 5, calculated for a structure with six cores equally spaced in a ring. The azimuthal component of power flow is proportional to the weighted sum over all OAM orders
3.11
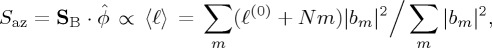

where 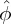 is a unit vector pointing in the azimuthal direction, **S**_B_ the Poynting vector of the Bloch wave and 〈*ℓ*〉 is the expectation value of the OAM. **S**_B_ points in the direction of the group velocity *∂ω*/*∂***k**, which is normal to the dispersion curves (when they are plotted isometrically) in the direction of increasing optical frequency. The modes with order *ℓ*^(0) ^= 0 and +3 carry very little total OAM, because the clockwise and anticlockwise components in the summation in equation (3.1) approximately cancel. One the other hand, the modes with principal order *ℓ*^(0) ^= ±1 and ±2 carry significant total OAM.

## 4. Topological effects

Interest in the propagation of electromagnetic waves in helical structures dates back at least to the 1940s, with the invention of the travelling wave tube amplifier [20]. In this device, a microwave signal is guided along a helical wire that spirals around an axially propagating electron beam. By appropriate design, the two waves can be velocity-matched, permitting the microwave signal to be amplified with power from the electron beam. This works by exploiting the third dimension, in the sense that the physical distance over which the spiralling wave travels is longer than the distance directly along the axis, i.e. both the group and phase velocities are effectively reduced.

The geometrical stretching of the PCF cladding structure, which increases quadratically with radius, has profound implications for the optical properties of the guided light. This is because, as mentioned above, it causes the effective optical path-length along the axis, and thus the effective refractive index, to increase topologically by the fractional amount
4.1
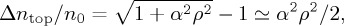

where *n*_0_ is the index in the untwisted case. This makes it possible, for example, to phase-match light guided in a central solid glass core (modal index *n*_c_) to the fundamental space-filling mode in the cladding (phase index *n*_SM_ in the untwisted fibre) at a radius given by
4.2
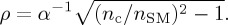


**Figure 6.**
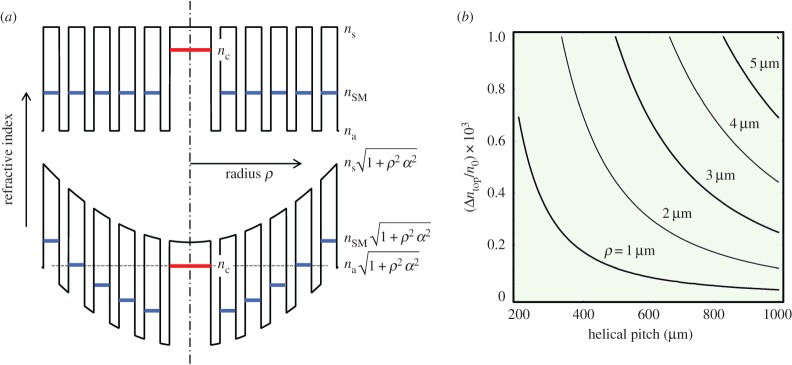
(*a*) Top: cut through the refractive index distribution of a solid-core PCF (schematic). The glass index is *n*_s_, the space-filling mode index is *n*_SM_, the index in the hollow channels is *n*_a_ and the core mode has index *n*_c_. Bottom: illustration of the effect of twist (schematic). The increase in path-length along the spiral paths raises the effective refractive indices by a factor (1 + *α*^2^*ρ*^2^)^0.5^, which makes it possible for the core mode to phase-match to the cladding mode at a certain value of radius. (*b*) Normalized topological index difference Δ*n*_top_/*n*_0_ (where *n*_0_ is the index in the untwisted fibre) versus helical pitch *L* for several different values of radius. (Online version in colour.)

As a result, light guided in the core can couple out into cladding modes, as illustrated in figure 6*a* (see also §5). Equation (4.1) is plotted in figure 6*b* for realistic values of twist rate and radius.

Twisting can have quite surprising effects in certain structures. For example, it was recently reported that a twisted core-less PCF can support low loss-guided modes, through a combination of twist-induced topological distortions and photonic band gap effects [21]. In another example, it was shown that the suppression of higher-order modes in single-ring hollow core PCFs can be optimized by twisting, once again through the topological distortion in equation (4.1) [22].

An unusual feature of helically twisted PCF with a single central glass core is a series of dips in its transmission spectrum, first observed experimentally [23]. These turn out to be caused by anti-crossings between the core mode and leaky cladding modes carrying OAM, each dip corresponding to a different OAM order [24]. Because the cladding light is diverted along a spiral path, the azimuthal component of its wavevector must take values that yield a round-trip phase advance that is an integer multiple *ℓ* of 2π, where *ℓ* is the OAM order. This yields the condition
4.3
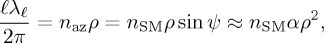


where *λ_ℓ_* is the dip wavelength of the *ℓ*th OAM order and *n*_az_ the azimuthal component of refractive index. Equation (4.3) yields remarkably good agreement with experimental measurements [24], showing in particular that the dip wavelengths scale linearly with the twist rate. Because the effective axial refractive index increases quadratically with radius (figure 6), light will refract outwards away from the axis, rendering the cladding resonances highly leaky; this causes the strong dips in transmission of the core light.

**Figure 7.**
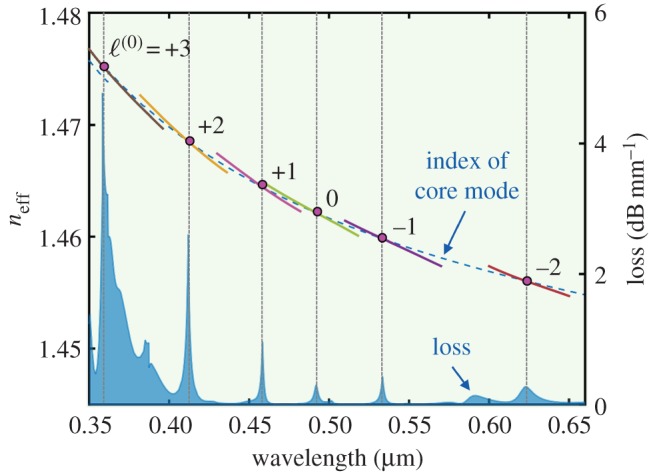
Effective index of the core mode of a twisted PCF, plotted against wavelength (blue dashed curve). The intersecting coloured curves show the effective indices of successive ring-shaped modes in a PCF with no central core. These modes carry principal OAM orders as indicated, and because they are also highly leaky, they cause strong core mode loss at the anti-crossing points (right-hand axis). The hollow channel diameter is 1** **µm, and the spacing is 3** **µm. The twist period *L**** ***=** **700** **µm. (Online version in colour.)

An intriguing aspect of equation (4.3) is that it does not contain any strict requirement that the core mode be phase-matched to a cladding mode at each dip. This presents a conundrum, for phase-matching would seem to be essential. Although the topological increase in index with radius makes it more likely that phase-matching can be achieved (figure 6), this does not yield a precise condition either. The explanation lies in the high loss of the cladding resonances, which widens the coupling bandwidth and relaxes the phase-matching condition. This has the additional consequence that the strength of the loss peaks will vary according to how well phase-matched the core mode is to the cladding resonance. This is confirmed by the numerical simulations in figure 7, where the effective indices *n*_eff_ and loss values of the cladding resonances and the core mode are plotted against wavelength, showing the anti-crossing wavelengths and relatively broad-band loss peaks.

**Figure 8.**
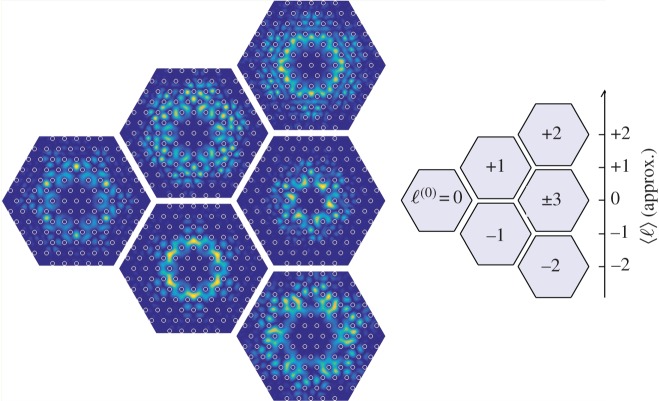
Axial Poynting vector distributions of the full set of six leaky ring-shaped cladding modes, calculated for a structure with no central core. The principal OAM order of each mode is indicated on the right, corresponding to the anti-crossings in figure 7. The approximate expectation value of the OAM, 〈*ℓ*〉, is also indicated. (Online version in colour.)

Each anti-crossing can be associated with a different principal OAM order, as indicated by the labels in figure 7. The axial Poynting vector distributions, associated with the ring-shaped modes at each anti-crossing, are plotted in figure 8. Because the fields are a superposition of several OAM orders *ℓ*^(0) ^+ *mN*, the expectation value of the OAM for each mode is not integral, but is given by the weighted sum over all the harmonics, as expressed in equation (3.11). Usually, one OAM order is dominant, however, except for *ℓ* = ±3 and *ℓ* = 0, when the weighted sum 〈*ℓ*〉 is approximately zero.

In the vicinity of the dips, it turns out that light couples to leaky OAM modes in the cladding at rates that depend on the polarization state. As a result, strong circular dichroism is seen [25]. This has been demonstrated experimentally by measuring the ellipticity angle of the output polarization state (for linearly polarized input) as a function of wavelength across a dip. The ellipticity angle first peaks strongly positive before changing sign and peaking to a negative value before eventually approaching zero (the peaks swap sign when the sign of the chirality is changed). This response is accompanied by enhanced optical rotation.

## 5. Optical activity in twisted photonic crystal fibre

The term optical activity derives from the interaction of chiral materials with polarized light. The (−) or laevorotatory form of an optical isomer rotates the plane of polarization of linearly polarized light anticlockwise. The (+) or dextrorotatory form of an optical isomer does the opposite.

A remarkable feature of the modes guided in twisted PCF is that, above a certain minimum twist rate that depends on the structural parameters, they are robustly circularly polarized. This is because the left (LC) and right (RC) circularly polarized modes have different propagation constants, resulting in optical activity (for linearly polarized light) and preservation of circular polarization state. The only situation when this is not the case occurs in the vicinity of the spectral dips discussed above, when the LC- and RC-polarized ring-modes have different loss rates, resulting in circular dichroism. In this section, we explore the physics of optical activity in twisted PCF, which by virtue of its sixfold symmetry has zero linear birefringence [13].

### (a) Optimizing the circular birefringence

**Figure 9.**
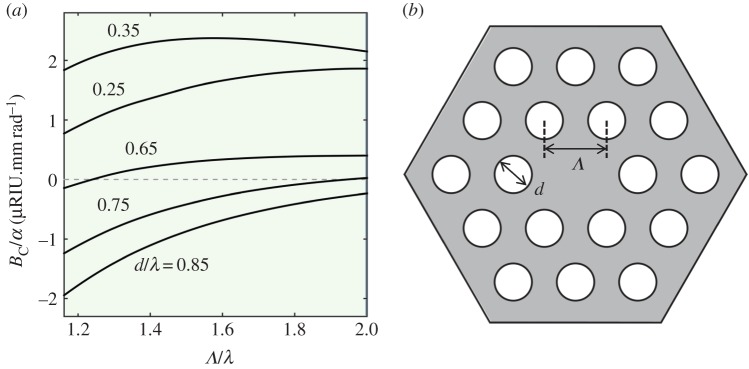
(*a*) Plot of *B*_C_/*α* versus scale parameter *Λ*/λ for different values of *d*/*Λ* (see right-hand sketch for the geometrical parameters). (*b*) The circular birefringence is maximum when *d*/*Λ**** ***=** **0.37 and *Λ*/λ** **=** **1.51. (Online version in colour.)

In a twisted PCF, the circular birefringence scales linearly with twist rate. For fibres made from fused silica, numerical modelling shows that *B*_C_ reaches a maximum for a shape parameter *d*/*Λ* of 0.37 and a scale parameter *Λ*/λ of 1.51 (figure 9). This has recently been confirmed experimentally by testing a range of different structures [26]. The physical reason for this behaviour lies in the modal field structure. When *d*/*Λ* approaches its maximum value of 0.5, the core boundary is only slightly hexagonal, reducing the effect of the twisting on the light. When *d*/*Λ* is very small, however, light scattering by the hollow channels is very weak, and once again, the effect of twisting is small. The optical activity is strongest when the modal ‘spokes’ that point out between the hollow channels have maximum visibility; this turns out to occur at *d*/*Λ *= 0.37.

### (b) Optical activity in spiralling off-axis cores

**Figure 10.**
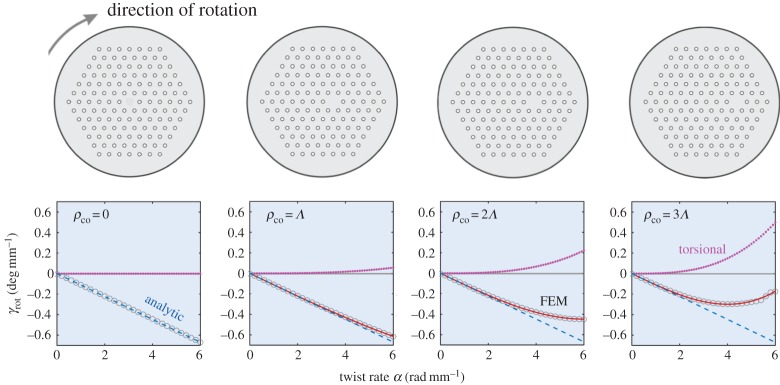
Rotation rates of linear polarization in four twisted silica PCFs (illustrated at top) with different off-axis core distances (*d*/*Λ*** **=** **0.33 and λ/*Λ*** **=** **0.27). Positive rotation rates signify that linearly polarized light rotates in the same direction as the twist. A twisted step-index fibre with an off-axis circular core yields a positive rotation rate (the violet curves in the bottom panels), whereas an on-axis core in a twisted PCF produces negative optical rotation, linearly proportional to the twist rate (dashed blue lines) [12]. The net value of *γ*_rot_ (equation (5.2)), therefore, follows the red curve, in excellent agreement with numerical simulations (circles). At values of *α* greater than 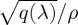 torsional rotation dominates. (Online version in colour.)

It was shown in the 1980s that a fibre with step-index core, circular in cross section and made from an isotropic homogeneous material, displays optical activity when it is helically coiled. The resulting rotation of the direction of linear polarization in the laboratory frame is a consequence of the constant torsion along the helical trajectory [27] and is a purely geometrical effect [28]. It gives rise to a rotation of the plane of linear polarization at a rate (in radians per unit distance) given by
5.1
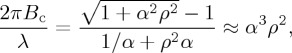

in the same direction as the twist (travelling along the fibre; figure 10). Another way to interpret this effect is to note that LC and RC polarized light have different reflection coefficients at the helically curved core-cladding boundary, giving rise to different propagation constants for LC and RC modes, i.e. circular birefringence *B*_C_. For an on-axis PCF core, it turns out that the plane of linear polarization rotates against the twist in proportion to *α* (dashed blue lines) [12]. The net optical rotation rate *γ*_rot_ for an off-axis PCF core therefore takes the form
5.2
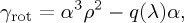

where *q*(λ) is a wavelength-dependent parameter that depends on the PCF structure (*q* = 0.002 in this case). In figure 10, *γ*_rot_ is plotted as a function of twist rate for four different silica PCFs with *d*/*Λ* = 0.33 and λ/*Λ* = 0.27, and a single core placed at *ρ*_co_ = 0, *Λ*, 2*Λ* and 3 from the axis. For an off-axis step-index core *q* = 0 and *γ*_rot_ depends on the third power of *α*, increasing quadratically with distance from the axis (violet curves). For a PCF with an off-axis core, however, these two effects compete, resulting in the red curves in figure 10. Comparisons with finite-element modelling show very good agreement, and it is interesting to note that *γ*_rot_ reaches zero at 
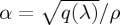
, turning positive for higher values of *α* when the torsional term begins to dominate.

## 6. Fabrication of twisted photonic crystal fibre

**Figure 11.**
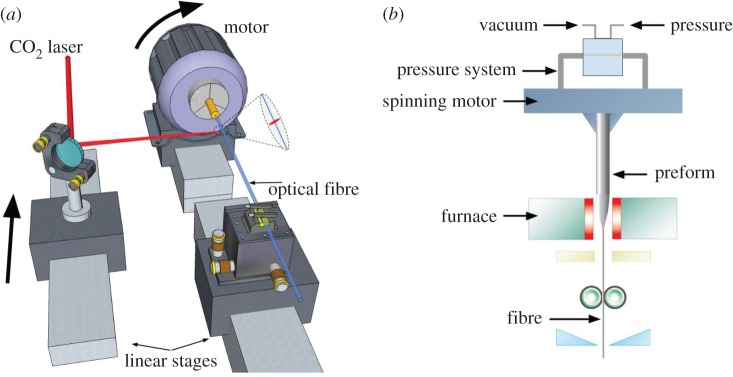
Sketches of the two fabrication procedures. (*a*) Thermal post-processing, using a scanned CO_2_ laser beam to heat the fibre to its softening temperature while spinning one fibre end, the other being rigidly fixed. (*b*) Spinning the fibre preform during the drawing process in the fibre pulling tower. (Online version in colour.)

Two main techniques are used for manufacturing twisted PCFs: thermal post-processing of an untwisted fibre, and spinning the preform during fibre drawing (figure 11).

### (a) Post-processing

A permanent twist can be imposed on an untwisted PCF by post-processing under CO_2_ laser heating (figure 11*a*). The fibre is mounted between a motorized rotation stage and a rigid support. As the motor rotates, the focused 10 µm laser beam is scanned along the fibre, using a steering mirror fixed to a precision motorized translation stage. A cylindrical lens is used to focus the light so as to reduce the sensitivity to laser beam misalignment. The laser power and the exposure time are adjusted, using a combination of a galvanometer-based variable attenuator and a shutter, placed in the path of the CO_2_ laser beam. Once the target twist period and sample length are set, the laser power and the scanning speed are chosen so as to heat the fibre to the glass-softening temperature. The writing process is computer-controlled and is capable of achieving twist periods as short as 300 µm. The set-up is designed to allow *in situ* transmission measurements during the writing process. It is possible to create complex pitch variations along the fibre using this technique.

### (b) Preform spinning during fibre drawing

Longer lengths of continuously twisted PCF can also be fabricated by spinning the preform during fibre drawing (figure 11*b*). This entails the use of a motor rotating at a few thousand rpm, and a rotary joint with multiple inlets for controlling the pressure inside the hollow channels. The twist period of the drawn fibre then equals the fibre drawing speed (m s^−1^) divided by the rotation rate (Hz) of the preform. Twist periods of a few millimetres can be routinely achieved over 100 m fibre lengths. The microstructure can be precisely optimized by active pressure adjustment during the draw.

### (c) Twist-induced distortions

It is clear that even if a preform cane has a perfect structure, it will undergo systematic distortions during twisting and drawing. For example, the further away the hollow channels and interstitial glass strands are from the axis, the more stretched they will become. There may also be some overall shrinkage of the fibre diameter, but as this is anyway a feature of fibre drawing we will ignore it. It is important, however, to assess the effects of radially dependent stretching, which will increase the length of a cladding strand (initially *L*_s0_) by *δL* = *ϵL*_s0_ while volume preservation will cause its diameter (initially *d*_s0_) to shrink by a factor (1 + *ε*)^−1/2^. If OPL_t_ is the optical path-length including only *δL* and OPL_t + s_ includes both *δL* and shrinkage, an estimate of the relative effects of shrinkage and stretching yields
6.1
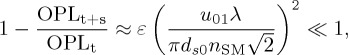

where *u*_01_ the first zero of J_0_ and *n*_SM_ the index of the fundamental space-filling mode in the straight fibre. For typical experimental parameters, equation (6.1) yields approximately 0.004*ε*, showing that the effects of shrinkage may be neglected.

## 7. Applications

### (a) Strain and twist sensing

The spectral dips discussed in §4 can be used to measure twist and strain [29]. Pure axial torsion will change the twist rate and shift the dip wavelength. On the other hand, if the fibre is subject to axial tension, then multiple effects come into play: the twist rate will be reduced, because the fibre gets longer, the transverse dimensions will shrink, and the photoelastic effect will increase the modal refractive index. Analysis shows that the resulting shift Δλ_R_ in the dip wavelength λ_R0_ takes the form
7.1
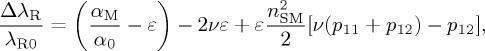

where *α*_0_ is the initial twist rate, *α*_M_ the imposed mechanical twist, *ε* the axial strain, *v* is Poisson's ratio, *n*_SM_ the index of the fundamental space-filling mode and *p_ij_* are the stress-optical tensor elements. Equation (7.1) suggests that twisted PCF can act as a transducer between torque and tension. If the fibre is twisted and stretched in such a way that Δλ_R_ = 0, then the mechanical twist and strain obey the relationship
7.2
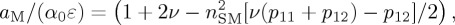

that is to say, if *α*_M_ is known then so is *ε*, and vice versa: a strain-twist transducer.

### (b) Current and magnetic field sensing

Interest in fibre-based current or magnetic field sensors dates back to the 1960s [30]. Such systems are attractive because of their compact size, immunity from electromagnetic interference, fast response time and wide operating temperature range [31–33]. They employ the Faraday effect, in which an applied magnetic field rotates the plane of polarization of linearly polarized light propagating along the optical fibre. For precise measurement of the Faraday rotation angle, a circularly birefringent fibre is ideal, because the output polarization state is then always exactly linear [34]. High values of *B*_C_ are highly desirable, because it is important that the circularly polarized modes are robust against external perturbations.

In earlier work on fibre-based current sensors, twisted linearly birefringent step-index fibre was used. The presence of linear birefringence meant that the twisted fibre did not exhibit pure circular birefringence [9]. This had the consequence that when linearly polarized light was launched, the polarization state at the output was elliptical. Although the ellipse axes did rotate when a magnetic field is applied, the current sensitivity, normalized to that in an ideal circularly birefringent fibre, was impaired by the factor
7.3
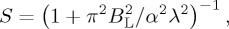

where *B*_L_ is the linear birefringence. Under these circumstances, it is also clearly more difficult to precisely measure the Faraday rotation angle than if the light is perfectly linearly polarized.

**Figure 12.**
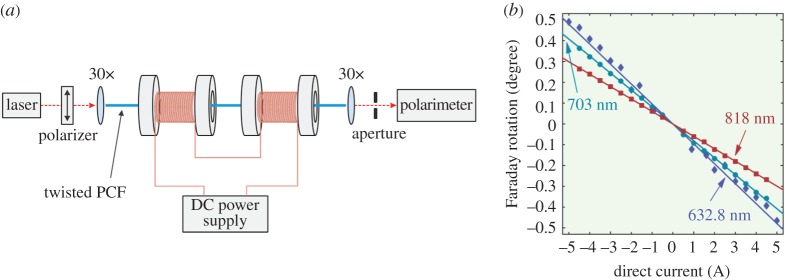
(*a*) Experimental set-up for measuring the Faraday rotation in an electromagnet. (*b*) Measured Faraday rotation plotted against current for three different laser wavelengths. (Online version in colour.)

The results of a recent experiment, using twisted PCF as a current sensor, are shown in figure 12. A 27 cm long twisted PCF (twist rate of *π* rad mm^−1^) was placed on axis at the centre of a solenoid and linearly polarized light launched into the fibre. The Faraday rotation angle, measured for three different wavelengths (632.8, 703, and 818 nm), is plotted against current and shows excellent linearity over the working range.

### (c) Orbital angular momentum-based telecommunications, optical tweezers

**Figure 13.**
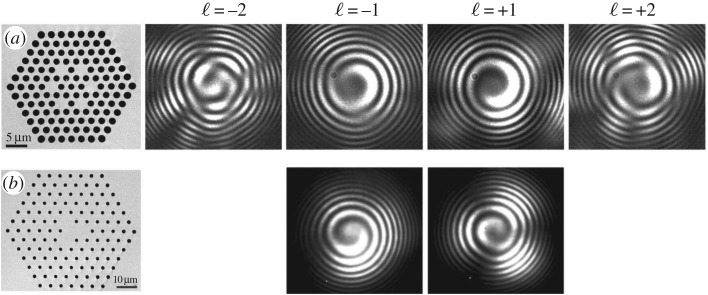
Left: scanning electron micrographs of twisted PCFs with (*a*) six and (*b*) three satellite cores. Four right-hand columns: experimentally recorded fringe patterns (formed by interference with a divergent Gaussian beam) of the different OAM-carrying modes in each case, after propagation along the twisted fibre (see text for more details).

The results of recent experiments on twisted PCFs with three and six cores arranged in a ring about the axis are shown in figure 13. The six-core PCF has hollow channels of diameter *d* = 2 µm, spaced by *Λ* = 3 µm, and the twist rate is *α* = 2.9 rad mm^−1^. For the three-core PCF, the values are *d* = 1 µm, *Λ* = 3 µm and *α* = 1.26 rad mm^−1^.

To determine the phase structure of the OAM modes after transmission through the twisted PCF, the output was superimposed onto a divergent Gaussian beam and the resulting fringe pattern imaged using a CCD camera (figure 13). For an approximately 1 m length of the six-core PCF, four helical Bloch modes with dominant OAM harmonics *ℓ* = ±1 and ± 2 could be independently excited by adjusting the launching conditions. The single- and double-helix interference patterns in figure 13*a*, which were recorded at a wavelength of 632.8 nm, confirm that the fibre generates optical vortices and preserves the magnitude and sign of the OAM for all four modes. Similar results were obtained at 1550 nm using the three-core PCF, which supports OAM modes with dominant order *ℓ* = ±1 (figure 13*b*). Similar experiments carried out at multiple wavelengths and for fibre lengths up to 50 m confirmed that the twisted PCFs preserve the magnitude and sign of the OAM.

The OAM birefringence displayed by twisted multicore PCFs is of interest in OAM-based optical communications [35,36] and may also be useful for flexible transmission of OAM in laser tweezer experiments for manipulation, for example, of cells.

### (d) Spectral and modal filters

Fibres with a straight central core surrounded by a ring of one or more spiralling satellite cores have been used to strip off higher-order modes in fibre lasers, rendering the core effectively single mode [37]. The spectral dips that appear in twisted single-core PCF can be used to form effective wavelength filters, the characteristics of which can be tuned by varying the twist rate along the fibre. Potential applications include suppression of unwanted spontaneous emission or lasing in fibre lasers and amplifiers. By appropriate use of circular dichroism or OAM birefringence, it may be possible to design lasers that emit pure circularly polarized beams carrying OAM.

## 8. Conclusion

The helically curved space within the periodic microstructure of a twisted PCF supports a family of unusual and fascinating effects, related to the ability of the spiralling structure to create optical vortices, i.e. act like an optical impeller. Twisting can create low loss guidance of light in a perfectly periodic and core-less PCF—the first example of a waveguide without a core. The modes of twisted PCF are almost perfectly circularly polarized, exhibit optical activity and carry OAM. They also display OAM birefringence, i.e. modes with principal OAM orders ±*m* have non-degenerate propagation constants. This means that the OAM can be robustly preserved over long fibre lengths. Twisted solid-core PCF exhibits a series of transmission dips at twist-tunable wavelengths, which have potential applications in sensing and filtering. Twisted hollow-core single-ring PCF offers enhanced elimination of unwanted higher-order guided modes [22]. It seems likely that many of these effects and phenomena will move into real-world applications in the near future. As yet unexplored is the use of twisted PCF in nonlinear optics and fibre lasers, where the combination of circular and OAM birefringence with control of group velocity dispersion may offer opportunities for new kinds of mode-locked soliton lasers, wavelength conversion devices and supercontinuum sources.
